# Hormone replacement therapy in morphine-induced hypogonadic male chronic pain patients

**DOI:** 10.1186/1477-7827-9-26

**Published:** 2011-02-18

**Authors:** Anna Maria Aloisi, Ilaria Ceccarelli, Maria Carlucci, Annalisa Suman, Gianfranco Sindaco, Sergio Mameli, Valentina Paci, Laura Ravaioli, Giandomenico Passavanti, Valeria Bachiocco, Gilberto Pari

**Affiliations:** 1 Department of Physiology, Section of Neuroscience and Applied Physiology, University of Siena, Siena, Italy; 2 San Carlo Clinic, Paderno Dugnano, Milano, Italy; 3 Pain Medicine Unit, Villa Serena Hospital and Advanced Algology Research, Forlì, Italy; 4 Pain Medicine Unit, ASL Cagliari, Cagliari, Italy; 5 Department of Urology "Misericordia" Hospital, Grosseto, Italy

## Abstract

**Background:**

In male patients suffering from chronic pain, opioid administration induces severe hypogonadism, leading to impaired physical and psychological conditions such as fatigue, anaemia and depression. Hormone replacement therapy is rarely considered for these hypogonadic patients, notwithstanding the various pharmacological solutions available.

**Methods:**

To treat hypogonadism and to evaluate the consequent endocrine, physical and psychological changes in male chronic pain patients treated with morphine (epidural route), we tested the administration of testosterone via a gel formulation for one year. Hormonal (total testosterone, estradiol, free testosterone, DHT, cortisol), pain (VAS and other pain questionnaires), andrological (Ageing Males' Symptoms Scale - AMS) and psychological (POMS, CES-D and SF-36) parameters were evaluated at baseline (T0) and after 3, 6 and 12 months (T3, T6, T12 respectively).

**Results:**

The daily administration of testosterone increased total and free testosterone and DHT at T3, and the levels remained high until T12. Pain rating indexes (QUID) progressively improved from T3 to T12 while the other pain parameters (VAS, Area%) remained unchanged. The AMS sexual dimension and SF-36 Mental Index displayed a significant improvement over time.

**Conclusions:**

In conclusion, our results suggest that a constant, long-term supply of testosterone can induce a general improvement of the male chronic pain patient's quality of life, an important clinical aspect of pain management.

## Background

Awareness of the need to adequately treat pain, particularly chronic pain, is slowly increasing among patients and pain therapists. Opioids are among the most prescribed analgesic drugs but present several side effects such as nausea, itching, constipation and hypogonadism [1,2]. For hypogonadism, there is clear evidence in the literature for both humans [3-7] and experimental animals [8,9]. Symptoms and signs related to gonadal hypofunction and consequent testosterone depletion include peripheral effects (muscle hypotrophy, bone loss, anaemia, etc.) and severe and disabling central effects (decreased attention, disappearance of libido, depressive state, etc.). Moreover, there is much evidence for a close relationship between testosterone and neuroprotective processes. For instance, it was reported that older men with low levels of free circulating testosterone appear to be at higher risk of developing Alzheimer Disease than men with higher levels of this hormone [10], and gonadal and adrenal androgen levels were found to be lower in men and women suffering from rheumatoid arthritis than in healthy controls [11]. In experimental animals, testosterone administration in inflammatory painful conditions, such as formalin- or adjuvant-induced pain, was found to decrease pain-induced responses [12-14].

Opioid-induced hypogonadism in males (Opioid-Induced Androgen Deficiency, OPIAD) is usually ignored by pain physicians and rarely considered for treatment [1] despite its high frequency (almost 100%) and persistence. Daniell [1] described the beneficial effects of testosterone replacement in opioid-treated patients for six months.

The aim of the present study was to describe the time course/interactions between opioids and testosterone replacement therapy in a clinical sample of male chronic pain patients diagnosed with OPIAD and receiving morphine via the epidural route. This kind of patient was chosen to reduce variables due to the high numbers of opiates available, their dosage and combinations. Moreover, all these patients had been suffering from chronic pain for many years and their pain condition was considered severe and stable. To obtain a broad picture of the effects induced by this treatment, we considered different aspects using a multidisciplinary approach. A team of experts, namely an andrologist, a psychologist, the project co-ordinator and a local pain therapist, met with each patient monthly for one year to check the clinical and laboratory outcomes. Testosterone was replaced via a gel formulation able to restore and maintain testosterone levels [15] by virtue of the fact that its application on the skin prevents first liver clearance and allows steroids to be directly absorbed and stored in the stratum corneum. From this reservoir, testosterone or its metabolites (17-beta estradiol and DHT) are slowly released into the circulation over several hours. Testosterone and its related hormones (estradiol, DHT) were measured in the blood, together with cortisol as an index of adrenal activity. Pain intensity and its features were studied with the VAS, the Italian version of the McGill Questionnaire (QUID) and the Margolis method to determine the percentage of the body in pain. The andrological condition was studied with the dedicated questionnaire Ageing Males' Symptoms Scale (AMS), and different aspects of the psychological characteristics were evaluated through questionnaires able to study anxiety, depression and quality of life (POMS, CES-D and SF-36). The study was carried out for 12 months. The results suggest beneficial effects on the patients' quality of life.

## Methods

### Subjects

Male chronic pain patients referred to two Italian pain centres (Forlì, Cagliari) were asked to take part in this study. To obtain the most homogeneous group of patients possible, only male subjects suffering from non-oncological chronic pain and undergoing epidural morphine were considered; morphine treatment had to have been carried out for at least six months; hypogonadism was diagnosed for testosterone values less than 2-3 ng/mL in at least two determinations in the previous 3-4 months. Other inclusion criteria were the presence of clear symptoms indicative of hypogonadism (fatigue, depressed mood, decreased libido) and negative urological problems. Experimental procedures were carried out in agreement with the Code of Ethics of the World Medical Association (Helsinki Declaration) and were approved by the Local Ethics Committee.

Once included in the study, each subject met with the experimental team once a month for one year during the morphine pump refilling session and underwent clinical, psychological and hormonal evaluations. In particular, the following procedures were performed during the monthly meeting:

- Blood collection,

- Complete clinical evaluation by the pain therapist, including the administration of specific pain questionnaires for pain assessment (VAS, QUID, Area%),

- Psychological evaluation, including the administration of specific questionnaires for anxiety, depression and quality of life (POMS, CES-D, SF-36),

- Andrological evaluation, including manual exploration of the prostate and transrectal prostate echography when necessary. The patient was asked to complete a specific andrological questionnaire (AMS). The subject was also examined for any adverse effects that might be related to the gel, including local skin irritation. At each visit, the subject was given a one-month supply of testosterone gel, a hydroalcoholic compound containing 50 mg testosterone in 5 g gel in each sachet. So as not to introduce other variables, the dose of testosterone gel was maintained constant independently of the blood testosterone levels shown by the patient. The patient was asked to apply the gel on the skin of the upper arm/shoulder or abdomen each morning at the same time, alternating the site of application each day and not taking a shower until 5 hours after application. He received verbal instructions to record in a diary any change that may have occurred in his daily activities and physical and mental performances.

All procedures took about 2 hours per patient per month.

#### Questionnaires

All questionnaires were self-administered, but supported by the presence of the expert clinician.

### Pain assessment

A Visual Analogue Scale, **VAS **(0-100), was used [16] to estimate the current intensity of pain and the peak intensity during the previous week. VAS is a 10 cm horizontal line, anchored at the extremes by "no pain " and "worst pain possible".

The quality and intensity of the current pain experience was evaluated by the Italian Pain Questionnaire, **QUID **[17]. The QUID is a reconstructed Italian version of the McGill Pain Questionnaire (MPQ). It is a semantic interval scale consisting of 42 descriptors divided into four main classes: sensory, affective, evaluative, miscellaneous. The Pain Rating Index rank value (PRIr) for each dimension and for the whole experience (Pain Rating Index rank-Total [PRIr-T], given by the sum of all the rank values) indicate the quality and intensity of pain.

The percentage of body surface area in pain (**Area%**) was measured according the Margolis method [18]. It consists of a human front-back body map in which the patient marks the area in pain. Thus, the drawing gives the pain distribution and the percentage of body surface area affected by pain.

### Andrological and urological evaluation

The impact of treatment on the androgens-related quality of life was measured by the Ageing Males' Symptoms scale (**AMS**) questionnaire [19]. The AMS is composed of three subscales, measuring respectively: psychological, somatic and sexual symptoms. Items (n = 17) are scored on a 1 (absent) to 5 (very severe) Likert scale. The sum-score for each subscale is obtained by adding up the ratings of the items. The total score is the sum of the three subscale ratings. The higher the value, the worse the condition of the subject. The severity categories are: mild (27-36), moderate (37-49), severe (≥50).

### Psychological evaluation

To study psychological characteristics of the patients and their time course, the following questionnaires were administered:

Profile of Mood State (POMS). The **POMS **measures the current psychological state of the participant [20] and consists of 58 feelings rated on a 5-point scale. It comprises six subscales: Tension-Anxiety (T-A), Depression-Dejection (D-D), Anger-Hostility (A-H), Vigor-Activity (V-A), Fatigue-Inertia (F-I) and Confusion-Bewilderment (C-B). In each subscale, values higher (T-A, D-D, A-H, F-I and C-B) or lower (V-A) than 55 were considered significantly altered with respect to the normal population [21].

Centre for Epidemiological Studies Depression Scale (CES-D). The Italian version of **CES-D **[22] was used to determine the level of depression symptoms for research purposes [23]. The CES-D is a 20-item self-reported measure of symptoms, scored on a scale from 0 to 3 frequency ratings ("less than 1 day" to "most or all (5-7) days") for symptoms within the past week. Previous studies have verified that a score of 16 or above on the CES-D indicates clinically significant depressive symptoms [23].

Italian version of the **SF-36 **[24] questionnaire. SF-36 is a 36-item questionnaire measuring the patient's level of performance in eight health domains: Physical Functioning, Role Physical (role limitations caused by physical problems), Social Functioning, Bodily Pain, Mental Health, Role Emotional (role limitations caused by emotional problems), Vitality and General Perception of Health. Individual items are scored on a 0-100 standardized Likert scale. For each domain, including Bodily Pain, a higher score indicates a better quality of life. Two indices (Physical and Mental) based on pertinent subscale clustering summarize the respective functioning. Due to the critical conditions shown by most of the patients at the beginning of the observations, some of the SF-36 data related to physical evaluation were lost. Thus only the Mental Index (MI) was taken into consideration.

#### Hormones

Blood was collected between 8.00 and 10.00 am in one EDTA-added tube (5 ml to obtain plasma) and one empty tube (10 ml to obtain serum). In addition to general laboratory exams performed locally (including albumin levels), serum and plasma samples were taken to the Stress and Pain Neurophysiology Laboratory of the University of Siena, where the following hormones were determined at baseline (T0), 3 (T3), 6 (T6) and 12 (T 12) months: total testosterone (TT), free testosterone (fT), dihydrotestosterone (DHT), estradiol (E2), sex hormone-binding globulin (SHBG), cortisol (C). Prostate-specific antigen (PSA) was determined each month. These values were used to calculate bioavailable testosterone (BioT) through the website http://www.issam.ch/freetesto.htm, according to Vermeulen's formula [25].

**Total testosterone **(TT) was measured by RIA using a kit from RADIM (Pomezia, Italy). The cross reactivity of the antiserum coated in the tubes was 5.6% for DHT, 1.6% for androstenedione and lower than 0.1% for androstenediol, SHBG, estrone, DHEAS, estradiol. The lower limit of quantitation of TT measured by this assay was 0.017 ng/mL. The intra- and inter-assay coefficients were 1.5% and 7.8%, respectively, at the normal adult male range: 3.5-8.5 ng/mL in our laboratory.

**Free testosterone **(fT) was measured by RIA using a kit from Diagnostic Systems Laboratories (Webster, Texas, USA). The cross reactivity of the antiserum coated in the tubes was 0.35% for 19-nor testosterone, 0.21% for 17 alpha-methyltestosterone, 0.13% for 11-oxo-testosterone and non-detectable reactivity for DHT, DHEA, DHEA-S, progesterone, estradiol, corticosterone and other androgens. The lower limit of quantitation of fT measured by this assay was 0.18 pg/mL. The intra- and inter-assay coefficients were 4.5% and 7.9%, respectively, at the normal adult male range: 14.7-32.7 pg/mL in our laboratory.

**Sex hormone-binding globulin (SHBG) **was measured by IRMA using a kit from Diagnostic Systems Laboratories (DSL, Webster, Texas, USA). Concerning the specificity, no human serum protein is known to cross react with the antibodies employed in the DSL SHBG IRMA system. The lower limit of quantitation of SHBG measured by this assay was 3 nmol/L. The intra- and inter-assay coefficients were 2.7% and 10.2%, respectively, at the normal adult male range: 28-94 nmol/L in our laboratory.

**Dihydrotestosterone (DHT) **was measured by RIA using a kit from Diagnostic Systems Laboratories (Webster, Texas, USA). The cross reactivity of the antiserum coated in the tubes was 3.3% for androstandiol glucuronide, 0.6% for testosterone, 0.03% for androstandiol and no reactivity for androstenedione, estradiol, androsterone glucuronide, dehydroepiandrosterone, cortisol, deoxycortisol, 17 alpha-OH progesterone, progesterone. The lower limit of quantitation of DHT measured by this assay was 4 pg/mL. The intra- and inter-assay coefficients were 5.5% and 9.5%, respectively, at the normal adult male range: 250-750 pg/mL in our laboratory.

**Estradiol (E2) **was measured by RIA using an ultra-sensitive kit from Diagnostic Systems Laboratories (Webster, Texas, USA). The cross reactivity of the antiserum coated in the tubes was 2.4% for estrone, 0.21% for 17 alpha-estradiol and 16 keto-estradiol, 0.64% for estriol. The lower limit of quantitation of E2 measured by this assay was 2.2 pg/mL. The intra- and inter-assay coefficients were 6.5% and 9.3%, respectively, at the normal adult male range: 10.0-25.1 pg/mL in our laboratory.

**Cortisol (C) **was measured by RIA using a kit from RADIM (Pomezia, Italy). The present method has not shown cross reaction with the following steroids: estradiol, testosterone, prednisone, cortisone, corticosterone, deoxycorticosterone and 11-deoxycortisol. The lower limit of quantitation of serum C measured by this assay was 0.9 microg/L. The intra- and inter-assay coefficients were 4.9% and 7.9%, respectively, at the normal adult male range: 50-250 microg/L in our laboratory.

**Prostate-specific antigen (PSA) **was measured by IRMA using a kit from Diagnostic Systems Laboratories (DSL, Webster, Texas, USA). Ferritin, hCG, prolactin, atropine, flutamide, diethylstilbesterol, acetylsalicylic acid, caffeine, ibuprofen, indomethacin do not interfere with the measurement of PSA in the DSL-9700 ACTIVE^® ^PSA coated-Tube IRMA assay. The lower limit of quantitation of serum PSA measured by this assay was 0.013 ng/mL. The intra- and inter-assay coefficients were 4.6% and 9.8%, respectively, at the normal adult male range: 0.42-2.20 ng/mL in our laboratory.

### Statistical analysis

Data are expressed as mean ± SEM. Changes of the data across time were analysed by Friedman analysis of variance (ANOVA) and Kendall concordance. To better define any possible drug effect, we compared the values at each time of observation (T3, T6 and T12) with the basal level (T0) using the non-parametric Wilcoxon test. All analyses were performed with Statistica software. A level of P ≤ 0.05 was considered statistically significant.

## Results

In the six-month period devoted to patient selection, 25 outpatients referred to the pain clinics involved in the study and meeting the inclusion criteria were asked to participate; 17 of them met the enrolment criteria and signed a written informed consent form. The study design was prospective, pre-post analysis. All patients were affected by intractable non-cancer chronic pain treated for many years with different pharmacological regimens until the present when they received morphine (epidural route). In some instances, as in tetraplegic patients, morphine was associated with an adjuvant (baclofen, see Table 1). Neuropathic pain was the most common pain type, while pain from chronic pancreatitis was present in three subjects.

**Table 1:** Personal and clinical data of the selected male patients.

Patients	Age	Pathology	Therapy	Total testosterone basal (ng/ml)	
1	47	Hypertonic tetraplegia Spinal lesion	i.t. morphine + baclofen	1.18	
2	60	Chronic pancreatitis	i.t. morphine	0.8	
3	40	Lumbosacral radiculopathy	i.t. morphine + baclofen	2.94	
4	67	Lumbosacral neuralgia	i.t. morphine + marcaine	0.3	
5	58	Neurinoma	i.t. morphine	0.94	
6	44	Spinal surgery	i.t. morphine	1.05	
7	71	Lumbosacral radiculopathy	i.t. morphine	0.51	
8	68	Lumbosacral radiculopathy	i.t. morphine	1.4	
9	75	Coccygodynia	i.t. morphine	0.97	
10	43	Spinal lesion	i.t. morphine + durogesic	0.49	Interrupted after three months for personal reasons
11	64	Chronic pancreatitis	i.t. morphine	1.19	Interrupted after three months for personal reasons
12	55	Chronic pancreatitis	i.t. morphine	0.75	Interrupted for personal problems
13	74	Spinal lesion	i.t. morphine	1.16	Interrupted for personal problems
14	58	Myelopathy	i.t. morphine + baclofen	1.42	Interrupted for personal problems
15	72	Spinal lesion	i.t. morphine + baclofen	0.73	Urinary block
16	67	Myelopathy	i.t. morphine 3	2.1	Headache
17	79	Lumbosacral radiculopathy	i.t. morphine	1.94	Headache

Total testosterone values are reported to prove the hypogonadic condition. Reference range of total testosterone values: 3.5-8.5 ng/ml.

Of the 17 men included in the study (Table 1), three abandoned it within the first month and two after the third month for reasons independent of the study. In two subjects the immediate appearance of headache and in one subject the occurrence of urinary block obliged them to leave the study. The remaining 9 subjects (age 59.0 ± 4.4, range: 38-74) completed the 12 months of observation (Table 1). In these patients, it was not necessary to include any other treatment (i.e. physiotherapy, counselling) during the one-year study.

### Clinical observations

In all patients, treatment appeared to be immediately effective, as verbally confirmed during the interview by the patients and their relatives. Indeed, starting from the first month, patients reported general changes such as faster beard growth and increased appetite. Manual prostate exploration never revealed clinically relevant features and the PSA levels always remained within the physiological range (<3 ng/mL, mean 0.8 ng/mL).

### Morphine dose

**Figure 1:**
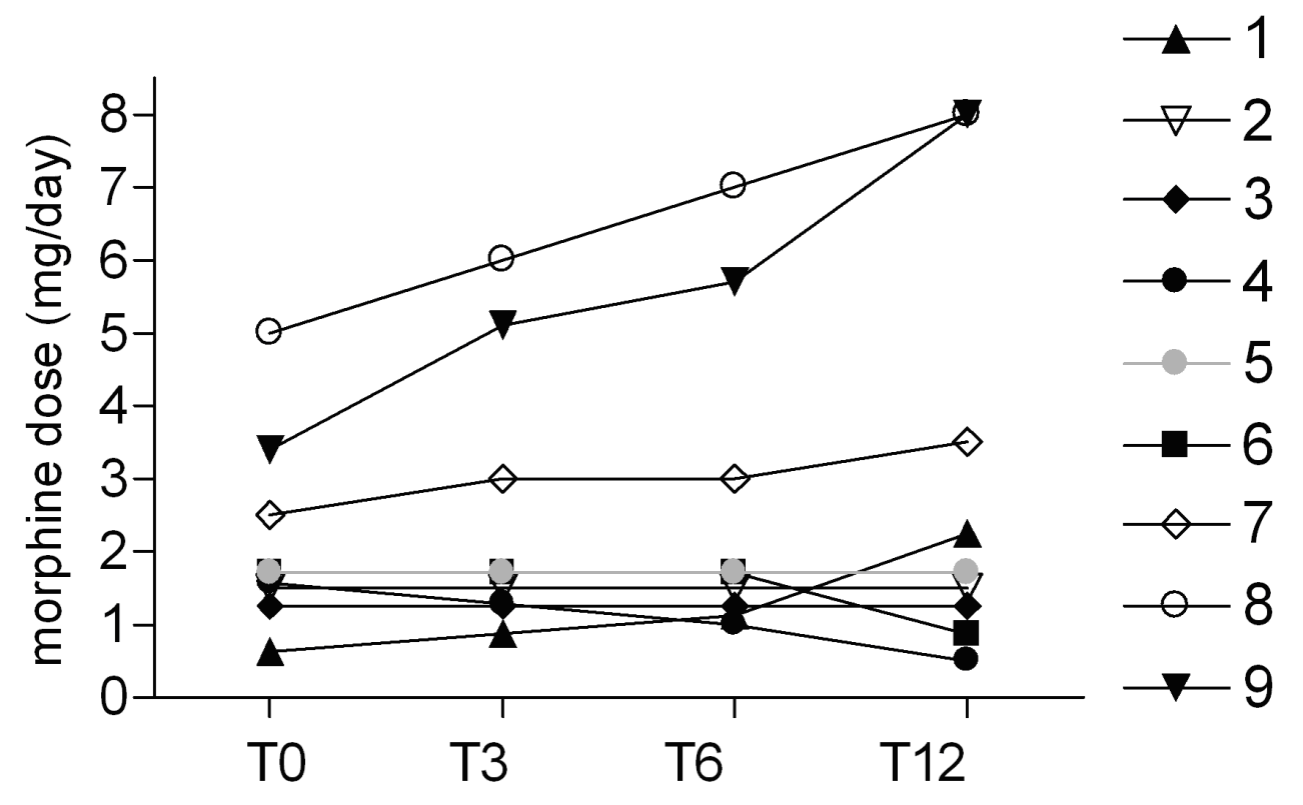
**Morphine dose assessment**. Time course of the morphine dose at basal time (T0) and after 3, 6 and 12 months of testosterone replacement therapy (T3, T6 and T12, respectively) in patients participating in the entire study. The lines represent the time course of the individual patients numbered as in Table 1.

The morphine dose was changed according to patient requirements (Figure 1). The dose remained stable in three patients, was decreased in two subjects and was increased in four. The last group included patients in which the improved general condition allowed a morphine increase in the attempt to complete the pain relief. This was made possible by the lesser number of side effects reported.

#### Pain assessment

##### VAS

Both VAS values (current and last week peak) were high and not modified by treatment; ANOVA did not show any significant variations (χ ^2 ^= 3.98, p = 0.26 and χ ^2 ^= 3.86, p = 0.27, respectively) (Table 2).

**Table 2:** Pain assessment

Parameter	*Months*			
	T0	T3	T6	T12
**VAS (0-100)**	67.14 ± 10.9	75.57 ± 6.44	66.42 ± 9.36	64.28 ± 9.15
**VAS last week (0-100)**	62.00 ± 11.15	82.85 ± 5.10	77.14 ± 6.80	72.14 ± 5.96
**Area %**	30.78 ± 10.03	29.04 ± 8.17	30.28 ± 7.77	31.00 ± 7.12
**QUID Sensory**	20.85 ± 1.86	14.57 ± 2.33*	12.85 ± 2.47#	16.00 ± 2.96
**QUID Affective**	11.14 ± 1.69	8.71 ± 1.71	7.57 ± 2.63	7.14 ± 2.07#
**QUID Evaluative**	17.28 ± 1.78	10.71 ± 2.75*	10.85 ± 2.82§	10.28 ± 2.92#
**QUID Miscellaneous**	11.71 ± 0.64	7.42 ± 1.81	6.14 ± 1.79§	7.00 ± 2.03
**QUID Total**	61.00 ± 5.06	41.42 ± 7.62*	37.42 ± 9.05*	40.42 ± 8.83*

VAS, Area% and QUID, values recorded before (T0) and after 3 (T3), 6 (T6) and 12 (T12) months of testosterone replacement therapy. Values are mean ± SEM (n = 9). Wilcoxon test: * p < 0.01 vs T0; # p < 0.02 vs T0; § p < 0.04 vs T0

##### Margolis

The percentage of body surface area in pain remained stable across time (ANOVA: χ^2 ^= 1.74, p = 0.63) (Table 2).

##### QUID

In all patients, the QUID values at T0 were quite high in total and partial ratings. Over time, there was a significant decrease in the PRIr-Total (χ ^2 ^= 13.87, p < 0.003) and in the sensory (χ ^2 ^= 8.29, p < 0.04), affective (χ ^2 ^= 8.06, p < 0.04) and evaluative PRIr (χ ^2 ^= 11.08, p < 0.01). The PRIr-sensory and PRIr-evaluative were already lower at T3 (p < 0.018 for both), the miscellaneous at T6 (p < 0.046) and the PRIr-affective at T12 (p < 0.046) (Table 2).

#### Andrological evaluation

##### AMS scale

The mean baseline score of AMS Total was 44.1 ± 4.1. This value indicates "moderate" andrological impairment. Detailed analysis of the subscale ratings revealed that there was a significant decrease, i.e. improvement, of the 'sexual' dimension (ANOVA: χ ^2 ^= 8.77, p < 0.03) (Table 3).

**Table 3:** Andrological assessment

Parameter	*Months*			
	T0	T3	T6	T12
**AMS Psychological**	11.00 ± 2.17	13.50 ± 2.67	9.28 ± 1.59	12.14 ± 1.95
**AMS Somatic**	15.57 ± 2.29	22.85 ± 2.25	20.85 ± 1.76	21.28 ± 1.91
**AMS Sexual**	14.14 ± 2.15	13.78 ± 1.85	9.71 ± 1.08*	10.42 ± 1.25*
**AMS Total**	44.14 ± 4.10	50.14 ± 5.63	39.85 ± 3.03	43.42 ± 4.00

AMS scale scores recorded before (T0) and after 3 (T3), 6 (T6) and 12 (T12) months of testosterone replacement therapy. Values are mean ± SEM (n = 9). Wilcoxon test: * p < 0.03 vs T0.

#### Psychological outcomes

##### POMS, CES-D and SF-36

All the basal values obtained in these questionnaires were outside the 'normal' ranges. In particular, POMS was higher than 55 in all subscales except Vigor-Activity (lower than normal), while CES-D was always higher than 16 and SF-36 lower than 50. None of the POMS subscale scores or CES-D ratings showed significant changes, while the SF-36 Mental Index displayed a significant improvement over time (ANOVA: χ ^2 ^= 11.35, p = 0.009); in particular, the score increased progressively to become significantly higher at T12 than at T0 (p < 0.04) (Table 4).

**Table 4:** Other questionnaires

Parameter	*Months*			
	T0	T3	T6	T12
**POMS Tension-Anxiety**	60.14 ± 6.14	65.71 ± 5.42	53.14 ± 4.86	58.71 ± 4.32
**POMS Depression-Dejection**	63.42 ± 7.16	70.28 ± 6.75	58.14 ± 6.69	65.00 ± 6.88
**POMS Anger-Hostility**	66.00 ± 7.45	68.14 ± 7.35	60.14 ± 4.36	61.85 ± 4.83
**POMS Vigor-Activity**	44.14 ± 3.90	37.28 ± 3.34	44.00 ± 3.50	42.28 ± 5.42
**POMS Fatigue-Inertia**	66.85 ± 7.25	72.00 ± 6.20	64.14 ± 6.42	64.56 ± 6.11
**POMS Confusion-Bewilderment**	60.85 ± 7.64	68.28 ± 7.66	58.28 ± 5.81	61.14 ± 7.22
**SF-36 Mental Index**	39.42 ± 5.98	35.00 ± 3.65	45.57 ± 4.33	48.42 ± 3.73*
**CES-D**	21.14 ± 6.55	27.85 ± 5.09	25.14 ± 4.71	22.71 ± 5.25

Scores of POMS, SF-36 and CESD questionnaires administered before (T0) and after 3 (T3), 6 (T6) and 12 (T12) months of testosterone replacement therapy. Values are mean ± SEM (n = 9). Wilcoxon test: * p < 0.04 vs T0

#### Hormonal parameters

Testosterone administration via gel is known to increase total testosterone serum levels immediately, reaching a steady state in 1-2 weeks [26]. Thus, the first result is that, although all our patients exhibited visible changes in body features (beard and hair increase) and in some habits closely dependent on androgens (increased food intake), the first significant increase in blood levels only occurred after 2 months of treatment as shown in the insert in Figure 1 where is depicted the time monthly time course recorded in the first 4 patients monitored to evaluate the efficacy of treatment.

### Total testosterone (TT), free testosterone (fT), bioavailable testosterone (BioT) and SHBG

**Figure 2:**
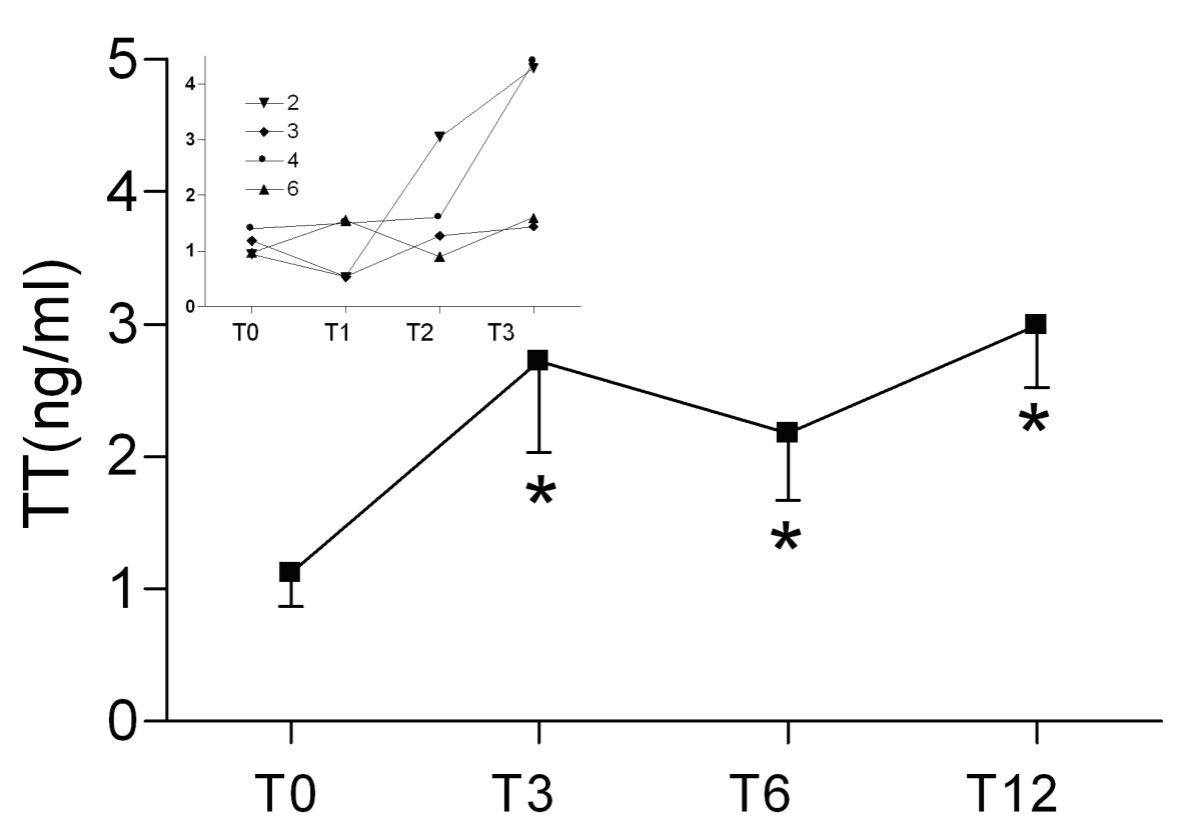
**Serum total testosterone**. Serum total testosterone (TT) values at basal time (T0) and after 3 (T3), 6 (T6) and 12 (T12) months of testosterone replacement therapy. The insert reports the TT levels at basal time (T0) and 1, 2 and 3 months from the beginning of testosterone replacement therapy (T1, T2 and T3, respectively) of the first four patients used to evaluate the time course of tissue androgen permeation. Data are mean ± SEM. * p < 0.05 vs T0.

Total (TT), free (fT) and bioavailable testosterone (BioT) showed very low levels at T0 (TT 1.16 ± 0.28 ng/mL; fT 4.33 ± 0.89 pg/mL, BioT 0.34 ± 0.1 ng/dL) with respect to the normal range, while SHBG was close to the upper limit of the normal range (77.77 ± 12.12 nmol/L) (Figure 2). Daily administration of testosterone significantly increased the TT serum levels at T3, remaining at approximately the same level until T12; there were similar results for fT, which became significant at T12 (p < 0.028). BioT was significantly increased at T3 (p < 0.028) and remained significantly higher at T6 (p < 0.05) and at T12 (p < 0.018).

**Figure 3:**
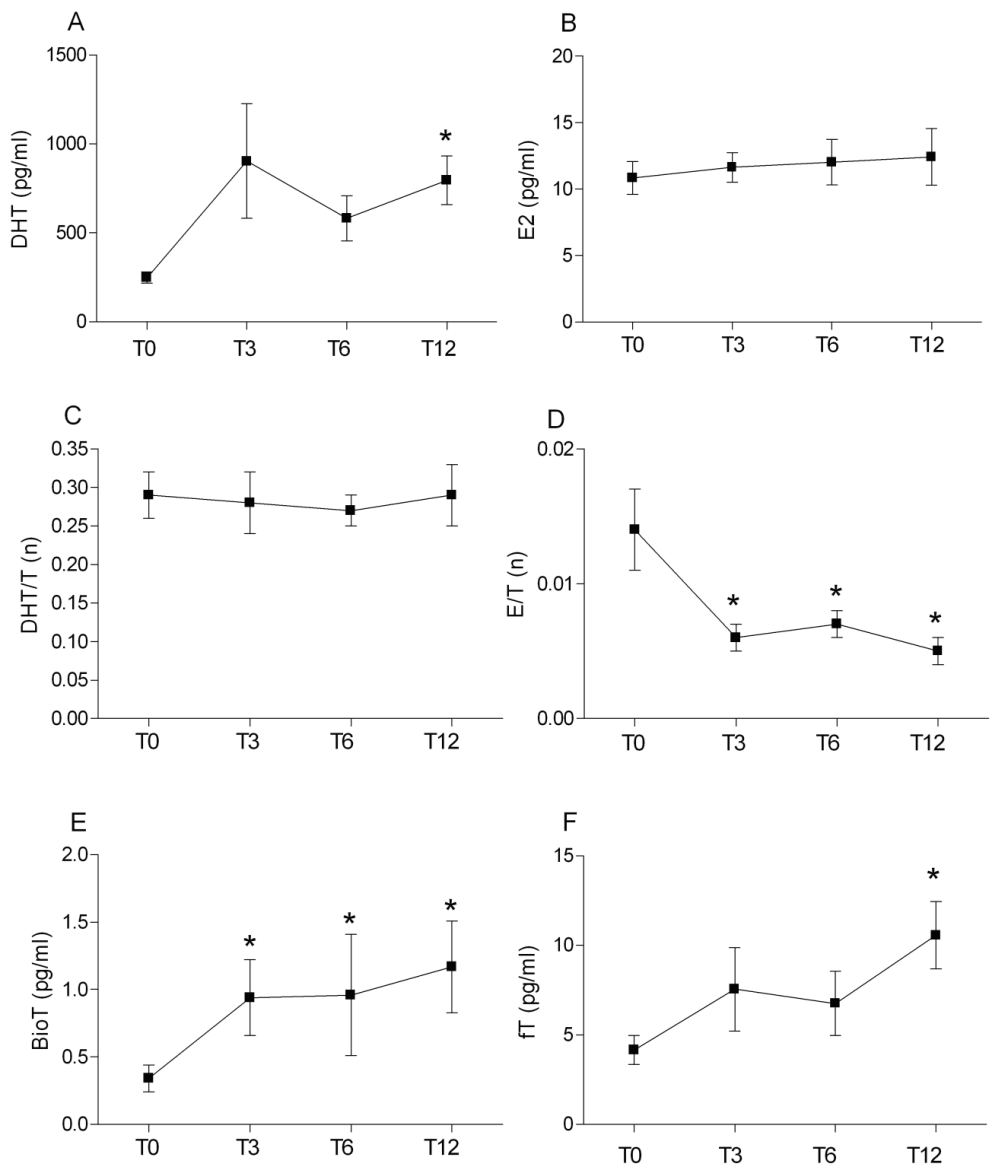
**Hormonal parameters evaluation**. Serum dihydrotestosterone (DHT, A), estradiol (E2, B), DHT/T ratio (C), E/T ratio (D), bioavailable testosterone (BioT, E) and free testosterone (fT, F) values at basal level (T0) and after 3, 6 and 12 months of testosterone replacement (T3, T6 and T12, respectively). Data are mean ± SEM. * p < 0.05 vs T0.

To evaluate the ratio between the unbound fraction (fT) and TT, we calculated a percentage (%fT). The %fT tended to decrease at T3, but did not reach significance. SHBG did not change significantly (Figure 3, Table 5).

**Table 5:** Hormonal parameters

Parameter (normal range)	*Months*			
	T0	T3	T6	T12
**TT (3.5-8.5 ng/mL)**	1.16 ± 0.28	2.72 ± 0.69**	2.18 ± 0.51*	2.99 ± 0.47**
**fT (14.7-32.7 pg/mL)**	4.33 ± 0.89	7.55 ± 2.34	6.76 ± 1.79	10.57 ± 1.88*
**BioT (ng/dL)**	0.34 ± 0.1	0.94 ± 0.28*	0.96 ± 0.45*	1.17 ± 0.34*
**SHBG (28-94 nmol/L)**	77.77 ± 12.12	89.41 ± 20.0	79.76 ± 15.7	54.15 ± 7.22
**% fT**	0.41 ± 0.04	0.31 ± 0.06	0.31 ± 0.02	0.36 ± 0.04
**DHT (250-750 pg/mL)**	248.65 ± 32.10	903.7 ± 321.9	582.45 ± 125.9	796.04 ± 137.22*
**E2 (10-25 pg/mL)**	10.84 ± 1.23	11.65 ± 1.09	12.03 ± 1.71	12.43 ± 2.13
**DHT/TT**	0.29 ± 0.03	0.28 ± 0.04	0.27 ± 0.02	0.29 ± 0.038
**E2/TT**	0.01 ± 0.003	0.006 ± 0.001**	0.007 ± 0.0007*	0.005 ± 0.001**
**C (50-250 ng/mL)**	164.04 ± 30.72	150.2 ± 22.1	131.58 ± 19.74	129.97 ± 15.57

TT: total testosterone, fT: free testosterone; BioT: bioavailable testosterone; SHBG: steroid hormone binding globuline; %fT: percentage of free testosterone; DHT: dehydrotestosterone; E2: estradiol; C: cortisol. Hormone levels determined before (T0) and after 3 (T3), 6 (T6) and 12 (T12) months of testosterone replacement therapy. Values are mean ± SEM (n = 9). Wilcoxon test: * p < 0.05 vs T0; ** p < 0.01 vs T0.

### Serum levels and ratios of the testosterone metabolites

The two metabolites of testosterone, DHT and E2, changed differently. DHT increased progressively, reaching significance at T12 (p < 0.02), whereas E2 did not vary. The ratio DHT/TT did not change while the ratio E2/TT was decreased significantly already at T3 and remained stable till T12 (p < 0.015, p < 0.021 and p < 0.011 at T3, T6 and T12, respectively).

Cortisol showed a slow, non-significant reduction during the one-year therapy (Table 5).

## Discussion

Evidence from this study suggests that one year of testosterone replacement therapy in male patients suffering from a severe form of chronic pain and diagnosed with morphine-induced hypogonadism (OPIAD) is able to positively change hormonal and behavioural 'indicators' chosen to measure different aspects of their condition.

Although pain research is continuously coming up with new products to treat chronic pain, opioids remain the reference therapy. Unfortunately this approach involves several side effects, which increasing morbidity would induce the discontinuation of therapy [27]. With continued opioid use, many side effects diminish or resolve; conversely, others such as immune alteration and hypogonadism persist and are even more apparent after long-term therapy [3,4,28]. The subjects considered in this study, all long-term opioid users, were also clearly suffering from hypogonadism, as revealed by the low plasma testosterone levels and/or clinical symptoms indicative of this condition.

Testosterone replacement therapy is a common approach in ageing males with partial androgen deficit or in young hypogonadic men due to traumatic or surgical causes; in these subjects, testosterone administration via gel is known to increase total testosterone serum levels immediately, reaching a steady state in 1-2 weeks [26]. Thus, it must be underlined that, although all our patients exhibited visible changes in body features (beard and hair increase) and in some habits closely dependent on androgens (increased food intake), the first significant increase in blood levels only occurred after 2 months of treatment. Further research is needed to better study the possible interaction among pain, opioid and androgen metabolism.

Several hypotheses have been advanced to explain OPIAD [29]. One suggests that the hypogonadism is due to opioid-induced inhibition of gonadotropin release [30]; however, another hypothesis suggests that the inhibitory action is also exerted in the gonads. Indeed, opioid receptors have been described in the pituitary as well as in the gonads, and opioids have been found to up-regulate their own receptors. Another mechanism involves testosterone metabolism. It is known that morphine increases 5-alpha reductase activity [9] and we have shown an excitatory effect on aromatase activity in vitro [31] and of both enzymes in ex-vivo tissues [32]; these enzymes, present in the liver but also widespread in body tissues, are involved in the transformation of testosterone to its metabolites dihydrotestosterone (DHT) and estradiol.

Finally, we cannot exclude either a direct effect of pain on the HPG axis, since observations have indicated a depressant effect of experimental pain on testosterone blood and brain levels in rats through the stress system, or the quite common use of other drugs able, like opioids, to inhibit the HPA axis [32,33].

### Andrology

While the psychological and somatic aspects of the painful condition are usually taken into consideration by physicians, the sexual aspect of the patient's life is generally disregarded. In the present study, as in a previous one by Daniell's group [1], the andrological questionnaires administered at the beginning of the observations confirmed the presence of altered conditions in all patients, probably related not only to pain but also to the altered hormone levels. Indeed also in the present study, the AMS revealed 'moderate' andrological disturbances, indicating the presence of clinically relevant alterations. As expected, testosterone replacement progressively improved the sexual aspect, as confirmed by the AMS's sexual dimension.

### Pain

We provide preliminary data suggesting that the pain experience, as assessed by QUID (a validated Italian pain questionnaire), is affected by testosterone replacement. In fact, QUID dimensions improved by the third month, reaching the best values at the sixth month. This was a very substantial result present in all subjects. QUID was more sensitive than the other pain measurement tools administered (VAS, %Area), probably because the questionnaire is composed of a series of descriptors requiring patients to carefully analyse and define the ongoing pain experience. Furthermore, since this instrument studies the different dimensions of the pain experience (i.e. sensory, evaluative, affective), we were able to verify that all these components benefited from the testosterone therapy, albeit following different time courses.

The morphine dose matched the change in pain shown by QUID. Although three subjects asked for escalating doses to complete the pain relief (also in virtue of the low side effects they experienced), the others maintained or reduced the morphine amount, thus implicitly expressing pain amelioration. The results of the SF-36 Mental Index agreed with these findings, as they indicated an improvement in the respective domains, i.e. daily activities, emotional competence and social relations. Of course, the improvement in these aspects may have positively influenced the pain perception and vice versa, since a virtuous circle was established.

The extension and distribution of the body surface area in pain remained unchanged, as did the VAS scores. From the beginning of the epidural morphine administration, all the patients complained that the last week before the refill was the worst of the month, even though the dose of morphine was repeatedly verified to be constant in the last days of the cycle. Our interpretation of the high VAS scores involves two mechanisms, the one involving patient attitudes and the other the instrument itself. Very long-lasting and unresponsive pain is not easily consciously modified since a feeling of helplessness is unavoidable in these subjects, impeding their realization that any change has occurred, especially when it is positive. VAS does not contrast this attitude. Due to its oversimplified nature, it does not require the patient to analyse and weigh his pain, and it facilitates recollection bias. In the end, the patient under-evaluates the measurement and over-emphasizes the pain he is rating. The findings on QUID, which requires the patient to focus on his experience, match perfectly with this explanation, making this instrument more reliable in the setting described herein. Interestingly these results are very similar to those reported by Daniell and colleagues (2006) [1], in which the general improvement of living conditions provided by patch testosterone replacement were not followed by a clear decrease in pain measures.

The affective state of chronic pain is characterized by deep depression and high anxiety; sometimes such an affective condition depends not only on the pain severity but also on the underlying disease, especially when it is highly vexing and disabling. Our patients were affected by intense long-lasting pain and a serious illness. Thus, they were anxious and depressed, as shown by the POMS and CES-D scores. These ratings did not vary throughout the observational period, indicating that testosterone therapy did not influence the patients' affective state. It is likely that the sense of uncontrollability of their condition was so deeply rooted in their mind that it was not easily attenuated. On the other hand, the improvement of pain was incomplete and too recent with respect to its history for the attitudes toward it to be changed. Furthermore, the patients knew that their disease was incurable. Interestingly the persistent high levels of anxiety and depression indicate that the decrease of pain shown by QUID cannot be attributed to the psychological component of the pain experience, as may occur in purposely treated chronic pain patients.

## Conclusions

The present results, although carried out in a small number of subjects and with a constant treatment dose, underline the possibility of successful testosterone replacement therapy in chronic pain patients treated with morphine. Hypogonadism is a usual consequence of opioid treatment, but it is rarely taken into consideration. Our results strongly suggest that this therapy can positively modulate the dimensions of pain. This effect allows us to propose the use of testosterone in clinics as an adjuvant, in combination with opioid therapy.

## Competing interests

The authors declare that they have no competing interests.

## Authors' contributions

AMA, IC, VB and GP conceived and supervised the project and edited the manuscript.

IC, GdP, MC, AS, GS, SM, VP, LR, GP participated in the experimental process and data analysis. All authors contributed to data interpretation. All authors read and approved the final manuscript.
